# PET Imaging of Small Extracellular Vesicles *via* [^89^Zr]Zr(oxinate)_4_ Direct Radiolabeling

**DOI:** 10.1021/acs.bioconjchem.1c00597

**Published:** 2022-02-28

**Authors:** Azalea A. Khan, Francis Man, Farid N. Faruqu, Jana Kim, Fahad Al-Salemee, Amaia Carrascal-Miniño, Alessia Volpe, Revadee Liam-Or, Paul Simpson, Gilbert O. Fruhwirth, Khuloud T. Al-Jamal, Rafael T. M. de Rosales

**Affiliations:** † Department of Imaging Chemistry and Biology, School of Biomedical Engineering and Imaging Sciences, King’s College London, St. Thomas’ Hospital, London SE1 7EH, U.K.; ‡ Institute of Pharmaceutical Sciences, School of Cancer & Pharmaceutical Sciences, King’s College London, Franklin Wilkins Building, London SE1 9NH, U.K.; § Electron Microscopy Centre, Department of Life Sciences, Faculty of Natural Sciences, Imperial College London, Flowers Building, London SW7 2AZ, U.K.

## Abstract

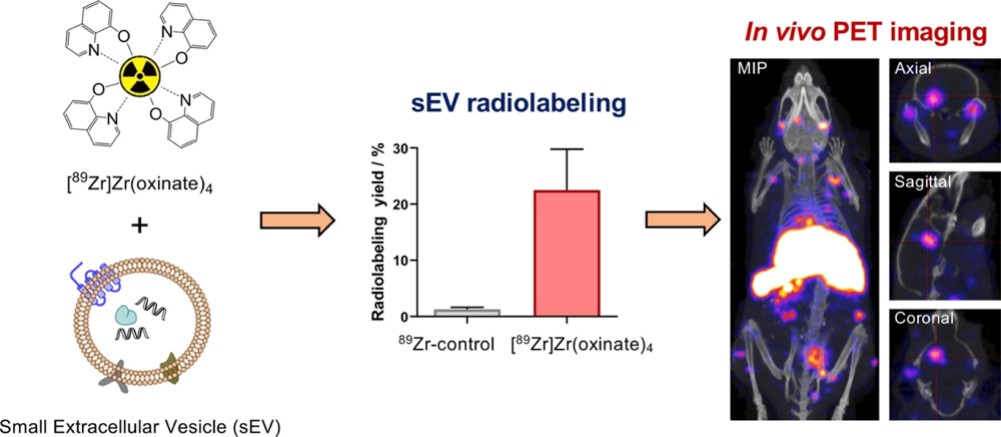

Exosomes or small extracellular vesicles (sEVs) are increasingly gaining attention for their potential as drug delivery systems and biomarkers of disease. Therefore, it is important to understand their *in vivo* biodistribution using imaging techniques that allow tracking over time and at the whole-body level. Positron emission tomography (PET) allows short- and long-term whole-body tracking of radiolabeled compounds in both animals and humans and with excellent quantification properties compared to other nuclear imaging techniques. In this report, we explored the use of [^89^Zr]Zr(oxinate)_4_ (a cell and liposome radiotracer) for direct and intraluminal radiolabeling of several types of sEVs, achieving high radiolabeling yields. The radiosynthesis and radiolabeling protocols were optimized for sEV labeling, avoiding sEV damage, as demonstrated using several characterizations (cryoEM, nanoparticle tracking analysis, dot blot, and flow cytometry) and *in vitro* techniques. Using pancreatic cancer sEVs (PANC1) in a healthy mouse model, we showed that it is possible to track ^89^Zr-labeled sEVs *in vivo* using PET imaging for at least up to 24 h. We also report differential biodistribution of intact sEVs compared to intentionally heat-damaged sEVs, with significantly reduced spleen uptake for the latter. Therefore, we conclude that ^89^Zr-labeled sEVs using this method can reliably be used for *in vivo* PET tracking and thus allow efficient exploration of their potential as drug delivery systems.

## Introduction

Exosomes, better described as small extracellular vesicles (sEVs), are cell-derived nanovesicles enclosed by a phospholipid bilayer, secreted by most cell types.^1^ They are formed inside endosomal multivesicular bodies and released into the extracellular space by exocytosis. sEVs are small in size (30–150 nm) and characterized by the presence of specific membrane-marker proteins such as CD63, CD9, Alix, and TSG101.^2^ The role of sEVs is the transport and exchange of cytosolic molecules (*i.e.*, nucleic acids, lipids, proteins, etc.) between cells,^3^ thus acting as messengers in cell–cell communication and disease progression.^4^ For example, tumor cell sEVs have been shown to promote tumor cell proliferation^5^ and metastasis^6^ and induce anticancer drug resistance.^7^ Interestingly, natural and drug-loaded sEVs (derived from stem cells, immune cells, or cancer cells) have shown therapeutic potential in cancer,^8^ Alzheimer’s disease,^9^ and type 2 diabetes.^10^ Furthermore, they have the ability to cross the blood–brain barrier (BBB)^11^ and to selectively target tissues.^12^ Therefore, there is an increasing interest in the use of sEVs as nanotherapeutics.^13^ In this context, it is important to develop imaging tools that track the *in vivo* behavior of sEVs. Doing so will improve our understanding of their biology and also support their development as drug delivery tools.

Optical imaging has been used to investigate the distribution of sEVs,^14^ but with associated challenges in quantification and signal tissue penetration. Radionuclide imaging can overcome these limitations. In particular, positron emission tomography (PET) imaging allows sensitive and quantitative whole-body imaging, with no background signal and unlimited tissue penetration in both animals and humans.^15^ At the time of writing, there are only a handful of peer-reviewed publications on the radiolabeling and *in vivo* imaging of sEVs,^16−30^ of which only three were aimed for PET imaging using three different radionuclides (^64^Cu, ^68^Ga, and ^124^I).^24−27^ These PET radiolabeling methods rely on the binding of radionuclides to membrane proteins which, given the importance of these surface components in the role of sEVs as messengers and cell–cell communication, may result in altered biodistribution and function as previously shown with ^111^In- and ^124^I-labeled sEVs.^16,26^ Consequently, radiolabeling within the intraluminal space of sEVs is desirable.

**Scheme 1:**
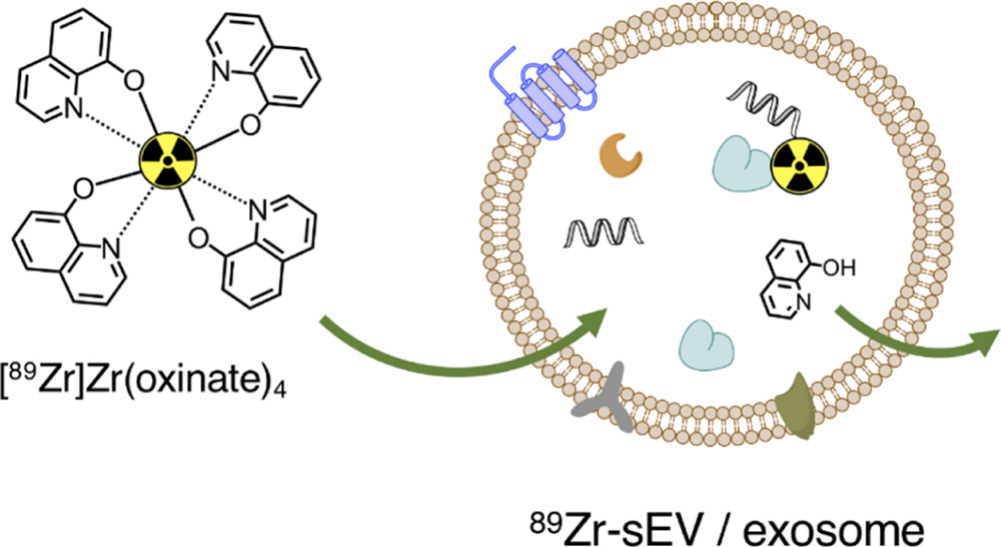
Schematic Representation of the Method for Intraluminal^89^Zr Radiolabeling of sEVs

Based on our previous work on cell and liposome radiolabeling,^31−33^ we hypothesized that radiometal complexes that are metastable, lipophilic, and neutral, such as those based on ionophore ligands, would allow intraluminal sEV radiolabeling (Scheme 1). In particular, the PET radionuclide ^89^Zr complexed by 8-hydroxyquinoline (oxine) allows direct radiolabeling of liposomes demonstrating intraluminal delivery of ^89^Zr across the lipid bilayer of vesicles.^31^ Here, we report a radiochemical synthesis method of [^89^Zr]Zr(oxinate)_4_ that allows efficient radiolabeling of sEVs and *in vivo* tracking using PET imaging.

The lipophilic [^89^Zr]Zr(oxinate)_4_ complex is able to pass through the lipid bilayer of the vesicles where ^89^Zr dissociates from the oxine ligands (that presumably become protonated and are able to cross the lipid bilayer), and ^89^Zr binds to intravesicular metal chelating ligands, such as proteins and nucleic acids, within the sEV.

## Results and Discussion

### Synthesis of [^89^Zr]Zr(oxinate)_4_

**Figure 1:**
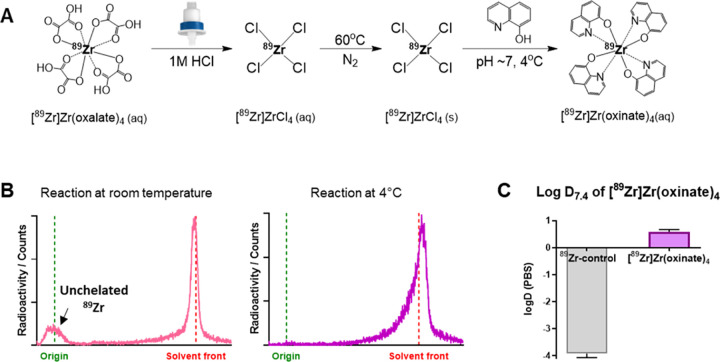
Synthesis and characterization of [^89^Zr]Zr(oxinate)_4_. (A) Schematic representation of the [^89^Zr]Zr(oxinate)_4_ synthesis. (B) Radiochromatogram showing presence of unreacted ^89^Zr when the reaction was performed at RT for 10 min but not when at 4 °C. (C) LogD_7.4_(PBS) of the ^89^Zr control and [^89^Zr]Zr(oxinate)_4_ synthesized at 4 °C (*n* = 3).

[^89^Zr]Zr(oxinate)_4_ synthesis was optimized for sEV radiolabeling (Figure 1A). In particular, the final solution had to be isosmotic to avoid sEV damage and with a high ^89^Zr concentration for *in vivo* PET studies. To achieve this, our synthesis involved the conversion of [^89^Zr]Zr(oxalate)_4_ in 1 M oxalic acid, as received from cyclotron production, into [^89^Zr]ZrCl_4_ (in 1 M HCl) by ion exchange chromatography.^34^ This was followed by a drying step involving gentle heating under a flow of N_2_ gas to remove HCl and H_2_O and allowing the concentration of the radioactivity. At this point, 80 μL of the oxine kit containing 1 M HEPES, 40 μg (0.3 μmol) of oxine, and 1 mg/mL polysorbate-80 at pH 7.8 was added (Method 1).^35^ Formation of [^89^Zr]Zr(oxinate)_4_ was confirmed using radiochromatography (Whatman No 1 cellulose as the stationary phase and ethyl acetate as the mobile phase). Using this system, [^89^Zr]Zr(oxinate)_4_ migrates to the solvent front (*R*_f_ = ∼1), whereas unreacted [^89^Zr]ZrCl_4_ stays at the origin (*R*_f_ = 0) (Figures 1B and S1A). Performing the reaction at 4 °C improved the radiochemical yield (RCY) compared to at room temperature (RT) (94.9 ± 2.1% *vs* 87.9 ± 5.7%; *p* = 0.0880; *n* = 4). Partition coefficient measurements (logD_7.4_) were consistent with the formation of a neutral lipophilic [^89^Zr]Zr(oxinate)_4_ complex (Figure 1C). [^89^Zr]Zr(oxinate)_4_ was also synthesized using an alternative method (Method 2) involving reaction of [^89^Zr]ZrCl_4_ with oxine as a solution in EtOH, followed by pH neutralization. No significant differences were observed between the two methods, based on RCY and logD_7.4_ assessments (Figure S1B). However, radiolabeling of sEVs using Method 1 was found to be highly reproducible and stable, hence was chosen for *in vivo* PET imaging studies.

### Isolation and Characterization of sEVs

**Figure 2:**
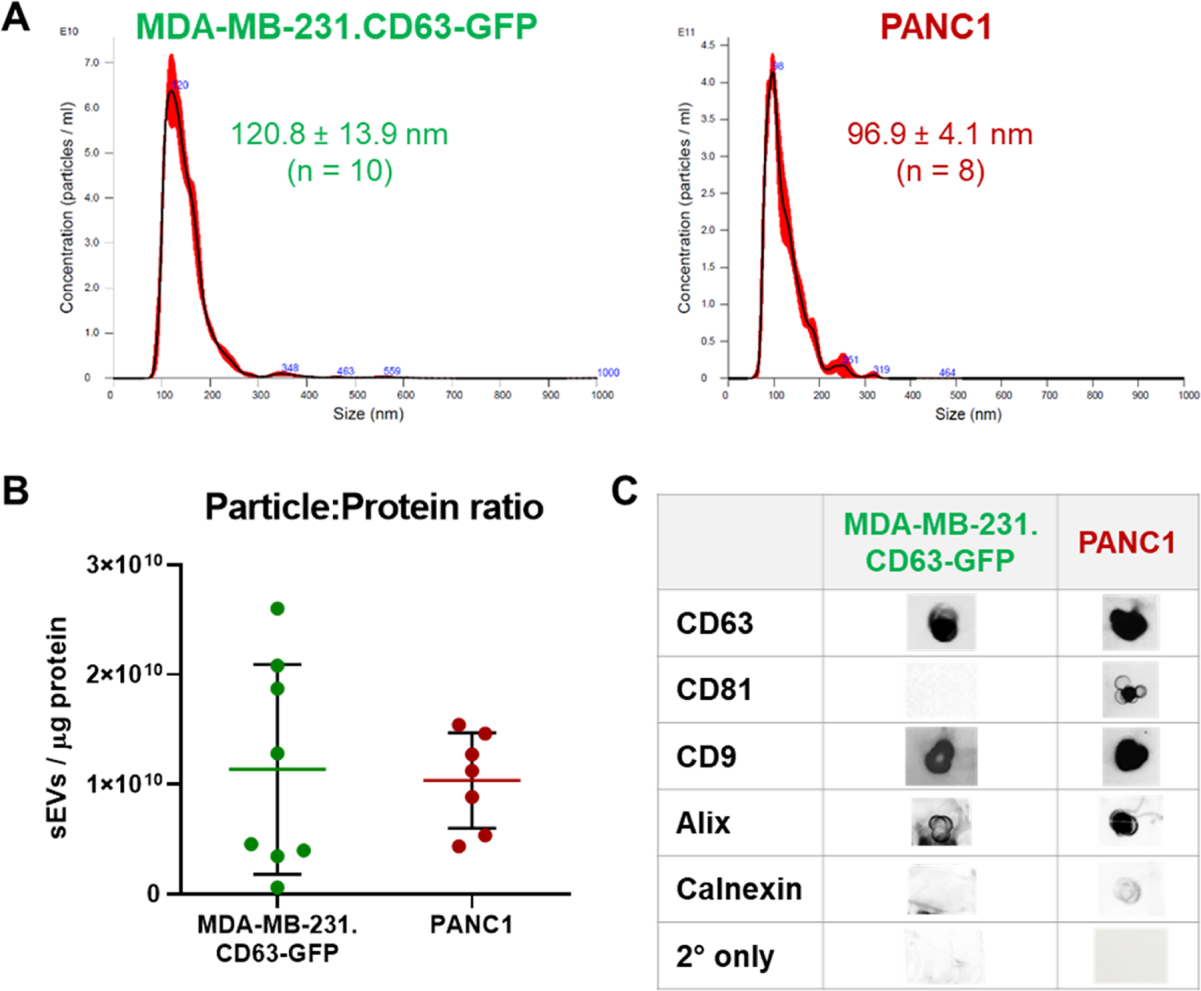
Characterization of small extracellular vesicles (sEVs). (A) Representative size distribution data from NTA for the two types of sEVs. Red areas represent the standard error of the mean of the triplicates (see Methods for details). The modal average hydrodynamic diameter of respective sEVs is shown; *n* = number of sEV isolations, data given as mean ± SD of the isolations. (B) Particle-to-protein (P:P) ratio of MDA-MB-231.CD63-GFP (*n* = 8) and PANC1 sEVs (*n* = 7), quantified by BCA protein assay; data given as mean ± SD. (C) Representative dot blots of MDA-MB-231.CD63-GFP and PANC1 sEVs.

As the release of sEVs from cancer cells is considerably higher than from normal cells,^36−38^ we isolated sEVs from the cell culture supernatant of two cancer cell lines (MDA-MB-231.CD63-GFP human breast cancer and PANC1 human pancreatic cancer cells) by differential ultracentrifugation. Nanoparticle tracking analysis (NTA) revealed that the average modal diameter for both sEVs was <150 nm, in compliance with the size range for sEVs, according to the Minimal Information for Studies of Extracellular Vesicles (MISEV) 2018 (Figure 2A). To determine the purity of the isolated sEVs, the particle-to-protein (P:P) ratio was measured. This ratio developed by Webber and Clayton^39^ determines the level of protein contamination in sEV samples, and a ratio < 1.5 × 10^9^ is considered “unpure”. A P:P ratio of > 1 × 10^10^ sEVs/μg protein was achieved for both MDA-MB-231.CD63-GFP and PANC1 sEVs (Figure 2B), indicating the purity of the isolated sEVs. Dot blot analysis of both sEVs demonstrated presence of sEV membrane markers CD63, CD81, and CD9, although CD81 was not detected on the MDA-MB-231.CD63-GFP sEVs. Furthermore, presence of Alix (endosomal protein) and absence of calnexin (endoplasmic reticulum-associated protein) indicated the endosomal origin (*i.e.*, definition of exosomes) and purity of the isolated sEVs (Figure 2C).

### Radiolabeling of sEVs with [^89^Zr]Zr(oxinate)_4_

**Figure 3:**
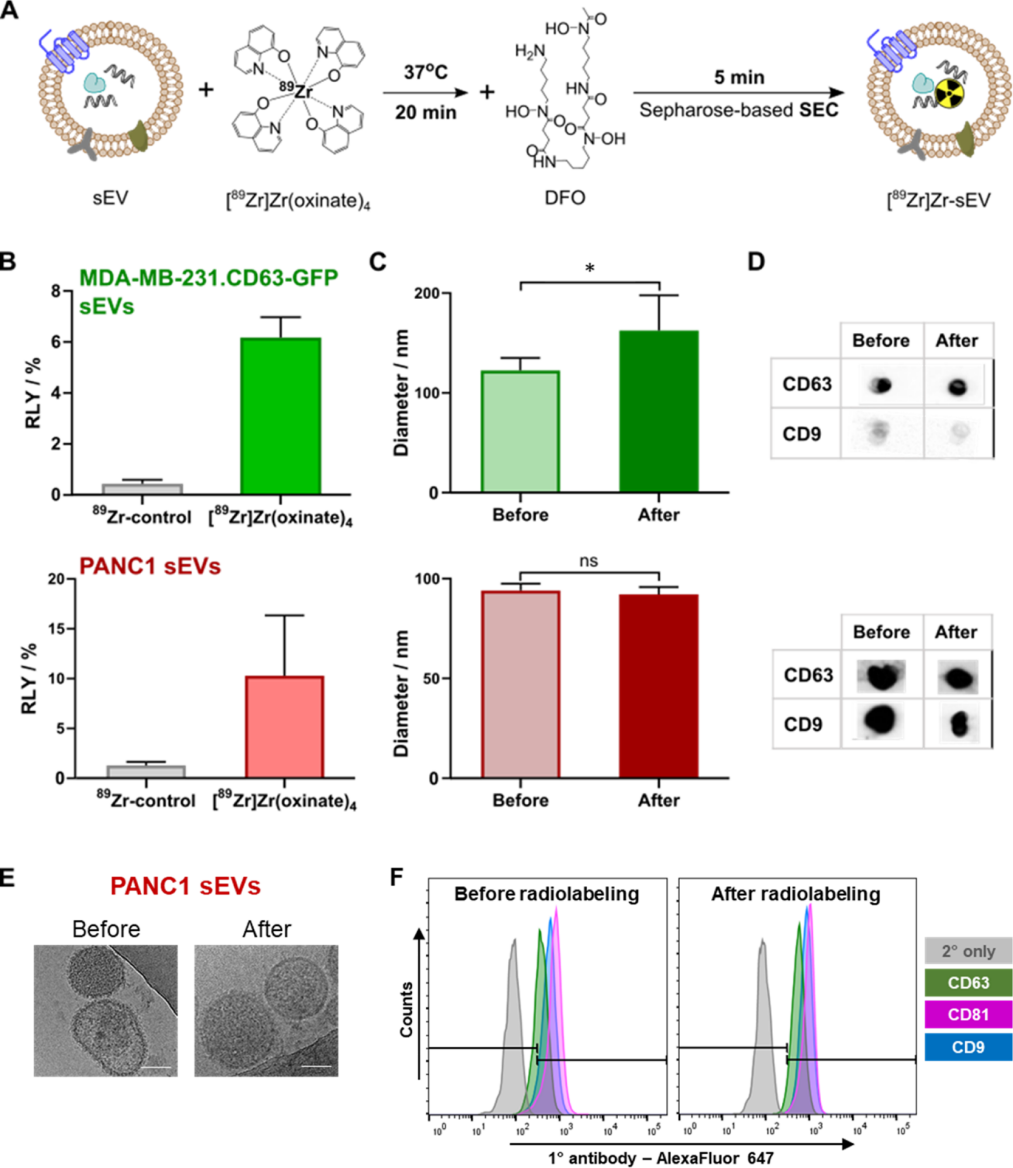
Radiolabeling and quality control of sEVs. (A) Schematic representation of the sEV radiolabeling protocol using [^89^Zr]Zr(oxinate)_4_. (B) RLY of 1 × 10^10^ MDA-MB-231.CD63-GFP sEVs (green) = 6.2 ± 0.8% and 1 × 10^11^ PANC1 sEVs (maroon) = 16.2 ± 4.0% (*n* = 3). (C) NTA data showing the hydrodynamic diameter of respective sEVs before and after radiolabeling, analyzed by Student’s unpaired *t*-test; * = 0.0138, ns = non-significant, and *p* = 0.05 was considered significant. (D) Presence of CD63 and CD9 proteins was detected on both MDA-MB-231.CD63-GFP and PANC1 sEVs by dot blot before and after radiolabeling. (E) CryoEM of PANC1 sEVs before and after radiolabeling; scale bar = 30 nm. (F) Histogram plot of bead-assisted flow cytometry analysis of PANC1 sEVs, showing no changes in the expression of the three sEV transmembrane proteins before and after radiolabeling.

We then tested the sEV radiolabeling capabilities of [^89^Zr]Zr(oxinate)_4_. sEVs were incubated with [^89^Zr]Zr(oxinate)_4_ for 20 min at 37 °C (Figure 3A). These conditions were chosen based on our previous studies showing that [^89^Zr]Zr(oxinate)_4_ cell radiolabeling is temperature-independent and rapid (<20 min).^32^ Following incubation, a small amount of the Zr chelator, desferrioxamine (DFO), was added to scavenge free ^89^Zr^4+^ ions from the reaction, including those that may be associated to the phospholipid membrane, as previously observed with liposomal vesicles.^40^ This ensures that ^89^Zr is only incorporated in the inside of the vesicles, by allowing efficient removal of any free or weakly bound extravesicular ^89^Zr *via* size exclusion chromatography (SEC). The same sEV radiolabeling procedure was performed using non-chelated ^89^Zr as a control (^89^Zr-control)—the same synthesis protocol and formulation as those of [^89^Zr]Zr(oxinate)_4_ but lacking oxine. The reaction mixture was then purified by Sepharose-based SEC systems that effectively separated sEVs from smaller molecules, including DFO-bound ^89^Zr (Figure S2). The results demonstrated significantly higher radiolabeling yields (RLYs) with [^89^Zr]Zr(oxinate)_4_ compared to ^89^Zr-control for both sEVs (Figure 3B), supporting our hypothesized radiolabeling strategy. Thus, [^89^Zr]Zr(oxinate)_4_—and not unchelated ^89^Zr—is able to pass through the lipid bilayer membrane into the intraluminal space of sEVs where ^89^Zr exchanges ligands and binds to intravesicular metal-chelating components, as we have previously demonstrated in cells and liposomes.^31−33^ Furthermore, the addition of DFO did not have any significant effect on sEV radiolabeling, suggesting that DFO neither enhances nor hinders the process (Figure S3A).

Tween-80, a common surfactant, is also present in the [^89^Zr]Zr(oxinate)_4_ formulation at a concentration of 1 mg/mL. The concentration of Tween-80 per radiolabeling reaction is ∼0.04 mg/mL, which is higher than its critical micellar concentration (0.02 mg/mL).^41^ Whereas this reagent is important to provide long-term *in vitro* stability to [^89^Zr]Zr(oxinate)_4_,^35^ it raises the concern that potential encapsulation of ^89^Zr by Tween-80 micelles may be involved in the sEV radiolabeling process. To exclude this possibility, we performed an experiment whereby an equal number of PANC1 sEVs were radiolabeled with [^89^Zr]Zr(oxinate)_4_ and their corresponding oxine-free ^89^Zr-control formulations, using both Methods 1 (containing Tween) and 2 (lacking Tween). The results showed that the presence of Tween-80 does not affect the RLYs of sEVs and hence that Tween is not involved in the radiolabeling reaction (Figure S3B).

There was no significant change in the hydrodynamic size of PANC1 sEVs before and after radiolabeling (*p* = 0.4754, *n* = 4), unlike MDA-MB-231.CD63-GFP sEVs (*p* = 0.0138, *n* = 4–8; Figure 3C). Despite detecting the sEV marker proteins CD63 and CD9 in both sEVs before and after radiolabeling (Figure 3D), the size instability of MDA-MB-231.CD63-GFP sEVs after radiolabeling prompted us to select PANC1 sEVs for further *in vitro* and *in vivo* experiments. There were no changes in the morphology of PANC1 sEVs, as analyzed by cryo-electron microscopy (cryoEM) (Figure 3E). Additionally, flow cytometry analysis of PANC1 sEVs’ membrane markers CD9, CD63, and CD81 pre- and post-radiolabeling further supports our hypothesis that intraluminal radiolabeling does not affect these membrane proteins (Figure 3F). This conclusion was reached because flow cytometry requires conjugation of beads to the sEVs, and thus their detection relies on intact vesicles (*vide infra*). However, further studies, such as proteomics, will be required to validate this. *In vitro* radiochemical stability was analyzed by instant thin-layer chromatography (iTLC) using 10 mM EDTA as the mobile phase to detect ^89^Zr^4+^ ions released from the vesicles, showing that ^89^Zr-PANC1 sEVs were 75.7 ± 3.4% (*n* = 3) stable after 26 h in phosphate-buffered saline (PBS) (Figure S4).

### *In Vitro* Cell Uptake of ^89^Zr-Labeled PANC1 sEVs

**Figure 4:**
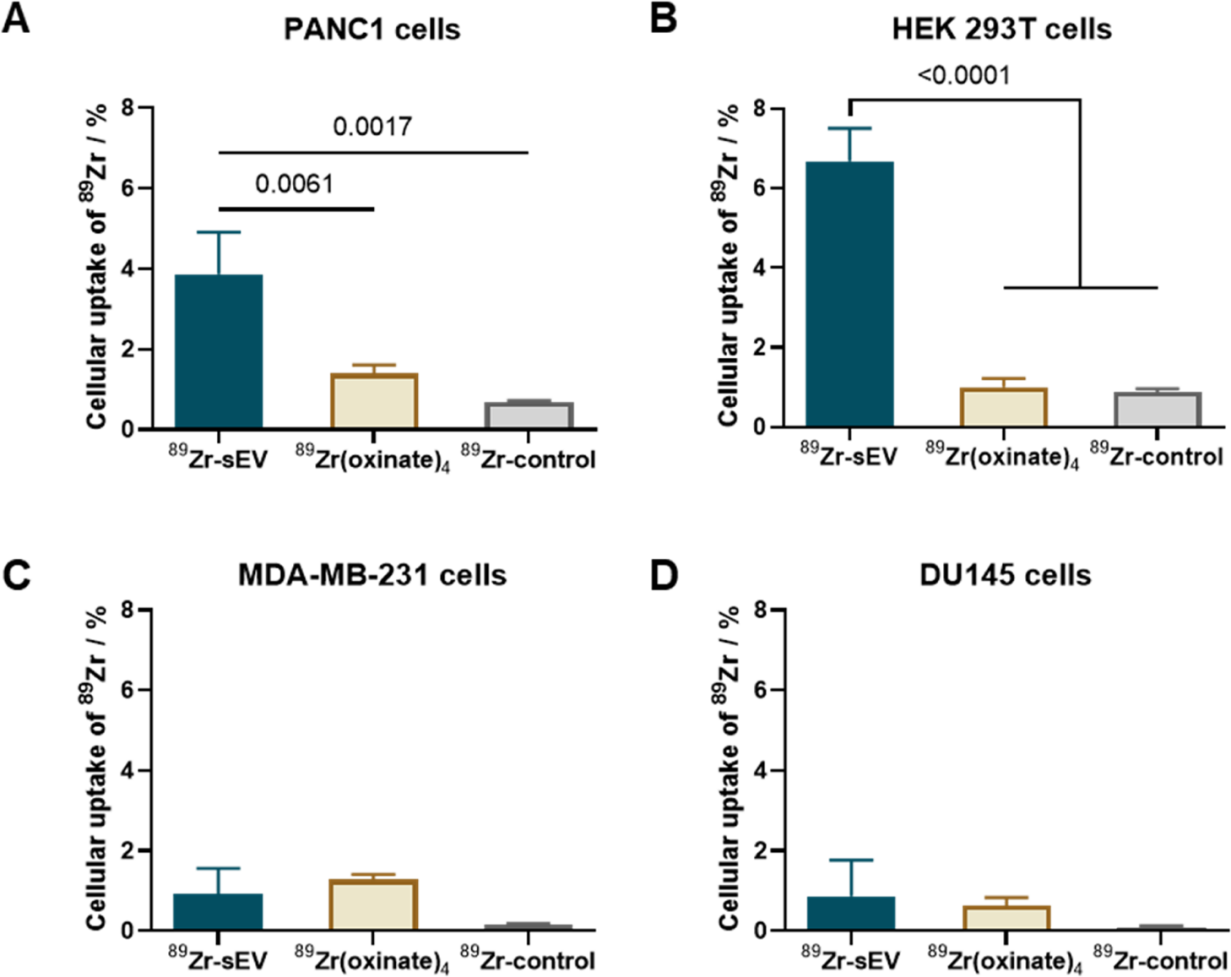
*In vitro* cell uptake of ^89^Zr-PANC1 sEVs. Cell uptake of ^89^Zr-PANC1 sEVs was analyzed in (A) PANC1 cells, (B) HEK 293T cells, (C) MDA-MB-231 cells, and (D) DU145 cells, after co-incubation in serum-supplemented media for 4 h. The final cell uptake data were normalized for 50,000 cells. Data are given as mean ± SD of *n* = 3 and analyzed by one-way ANOVA with Turkey’s correction for multiple comparisons.

Next, the ability of ^89^Zr-PANC1 sEVs to be taken up by different types of cells in serum-supplemented media was evaluated. The ^89^Zr-PANC1 sEVs, [^89^Zr]Zr(oxinate)_4_, and ^89^Zr-control were incubated at 37 °C with the following cells: PANC1 (parental cells), HEK-293T (healthy cells with known nanoparticle-uptake properties),^42^ MDA-MB-231, and DU-145 (non-parental cancer cells). Interestingly, ^89^Zr uptake by both PANC1 cells and HEK-293T cells was significantly higher for the ^89^Zr-PANC1 sEV group, compared to the two control groups (Figure 4A,B). In contrast, there were very low levels of ^89^Zr-PANC1-sEV uptake by the non-parental cancer cell lines (Figure 4C,D). It is worth highlighting the higher uptake of ^89^Zr-PANC1 sEVs in both PANC1 and HEK-293T cells compared to that achieved by [^89^Zr]Zr(oxinate)_4_, taking into account that the latter has proven cell-radiolabeling properties.^32^ Thus, these data demonstrate quick uptake of ^89^Zr-PANC1 sEVs by both parental cells and HEK-293T cells but not by other non-parental cancer cells.

### *In Vivo* PET-CT Imaging of ^89^Zr-PANC1 sEVs

**Figure 5:**
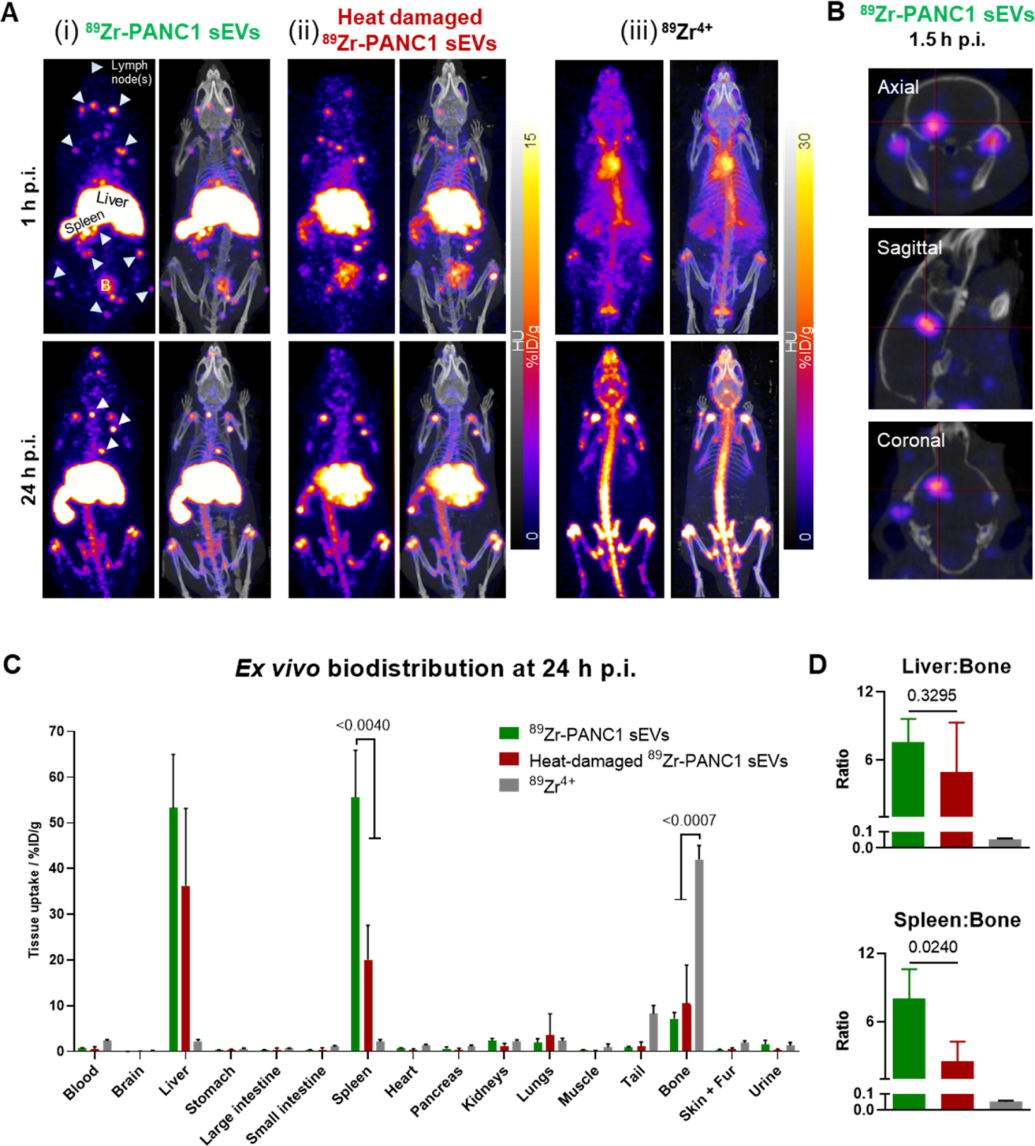
PET imaging and *ex vivo* biodistribution of ^89^Zr-PANC1 sEVs. (A) Maximum intensity projection PET-CT images of (i) intact ^89^Zr-PANC1 sEVs, (ii) heat-damaged ^89^Zr-PANC1 sEVs, and (iii) neutralized ^89^Zr^4+^ biodistribution in a C57BL/6j mouse at 1 h and 24 h post-intravenous injection; white arrowheads = representative LNs (see Figure S6) and B = bladder; the PET imaging scale for the ^89^Zr-control was adjusted for image clarity. (B) PET-CT images (axial, sagittal, and coronal slices) of a mouse injected with intact ^89^Zr-PANC1 sEVs showing uptake within the brain; the image scale is the same as in (A). (C) *Ex vivo* biodistribution showing uptake of “intact” (*n* = 4) and “heat-damaged” (*n* = 3) ^89^Zr-PANC1 sEVs and ^89^Zr^4+^ (*n* = 4); data given as mean ± SD. (D) Ratio of liver/bone uptake and spleen/bone uptake; data given as the geometrical mean ± SD. Statistical significances were calculated using Student’s unpaired *t*-test.

Encouraged by these results, we performed an *in vivo* PET-CT imaging and biodistribution study of PANC1 sEVs in healthy mice (C57BL/6). Immunocompetent healthy mice, and not diseased animals, were chosen as the best model to test our radiolabeling approach, as they provide a baseline for future applications of this radiolabeling methodology and allow direct comparison with other methods. Based on the *in vitro* stability studies (Figure S4), *in vivo* PET imaging was limited to 24 h, to minimize image/biodistribution analysis errors due to released free ^89^Zr. To assess the impact of damaged vesicles on the imaging of sEVs, we evaluated three groups: (i) intact ^89^Zr-PANC1 sEVs, (ii) heat-damaged ^89^Zr-PANC1 sEVs, and (iii) neutralized ^89^ZrCl_4_ (^89^Zr^4+^). The heat-damage protocol consisted of two cycles of heating and cooling (90°C to 0°C) ^89^Zr-PANC1 sEVs and was aimed at denaturing the vesicles but avoiding complete breakdown. Indeed, the heat-damage process resulted in an increase in size and partial release of internal contents (Figure S5A) and damage of sEV surface marker proteins compared to intact ^89^Zr-PANC1 sEVs (Figure S5B). ^89^Zr-PANC1 sEVs were prepared with a RLY of 32% (1 × 10^12^ sEVs). PET-CT imaging within 1 h post intravenous (iv.) injection (∼1 × 10^10^ sEVs/mouse) showed short circulation times and rapid uptake of intact ^89^Zr-PANC1 sEVs in the liver, spleen, bladder, several lymph nodes (LNs) [Figure 5A(i)], and brain [Figure 5B].

Short circulation times and liver/spleen/bladder uptake have been observed in other imaging studies of sEV biodistribution *via* iv. administration.^16−18,22,24,25^ However, to the best of our knowledge, this is the first time LN uptake is observed using *in vivo* imaging. With the help of CT imaging, the PET signals observed from the suspected LNs can be correlated with their well-documented location in mice (*e.g.,* cervical, brachial, pancreatic, renal, inguinal, popliteal, and others; Figure S6). sEV/exosome uptake in secondary lymphoid organs (*i.e.,* spleen and LNs) following iv. injection in the same mouse strain has been demonstrated and is mediated by CD169^+^ macrophages.^43^ Interestingly, sEVs are known to express α-2,3-linked sialic acid, which is the preferred ligand of CD169 thus providing a plausible explanation for the high spleen/LN uptake observed.^44^ It should be noted that not all mice showed clear LN uptake and hence was not possible to identify them and isolate them *ex vivo* for further analysis. The possibility of these imaging signals being due to released free ^89^Zr seems improbable due to its significantly different biodistribution [Figure 5A(iii),C]. In addition, intact ^89^Zr-PANC1 sEVs were visible within the brain (Figure 5B) but not in the heat-damaged ^89^Zr-PANC1 sEV group (Figure S7), supporting the previously reported ability of sEVs to cross the BBB.^11^ Heat-damaged ^89^Zr-PANC1 sEVs showed a similar biodistribution to intact ^89^Zr-PANC1 sEVs, with the major differences being a significantly lower spleen uptake and a higher bone signal [Figure 5A(ii)]. These two findings can be explained by the bigger size of the denatured vesicles and the partial release of contents we observed *in vitro* (*vide supra*), as a result of the heat-damaging process. In both groups, the bone signal increased at 24 h postinjection. This was expected and presumably due to the metabolic activity in the liver/spleen that will result in the release of bone-tropic “free” ^89^Zr. In addition, fewer LNs were visible, and no brain signal was observed.

The PET-CT imaging findings correlated with the *ex vivo* biodistribution data. Comparison of the intact ^89^Zr-PANC1 sEVs between 2.5 and 24 h suggests that once sEVs were taken up by the liver and the spleen, ^89^Zr remained in these organs, as no difference was observed in the liver and spleen signal between the two time points (Figure S8A). At 24 h post injection, a high liver/spleen signal and higher uptake of intact ^89^Zr-PANC1 sEVs in the spleen (55.7 ± 10.2 %ID/g) were observed, compared to heat-damaged ^89^Zr-PANC1 sEVs (20.1 ± 7.5 %ID/g), *p* = 0.0040 (Figure 5C and Table S1). The liver uptake was also higher for intact ^89^Zr-PANC1 sEVs, whereas the bone uptake was higher for heat-damaged ^89^Zr-PANC1 sEVs. From the *in vitro* stability study of intact ^89^Zr-PANC1 sEVs, we measured that ∼25% ^89^Zr is released from PANC1 sEVs over 24 h. ^89^Zr is a bone-tropic radionuclide and thus ^89^Zr released from the vesicles accumulates in the bone, as evident by the increased bone uptake from 3.6 ± 0.8 %ID/g at 2.5 h to 7.2 ± 1.3 %ID/g at 24 h (*p* = 0.0015, unpaired *t*-test; Figure S8A). This was also confirmed by the higher liver:bone and spleen:bone uptake ratio at 2.5 h (Figure S8B), compared to 24 h (Figures 5D and S8C). A differential uptake of intact *versus* heat-damaged ^89^Zr-PANC1 sEVs was observed for the spleen:bone uptake ratio (8.1 ± 2.6 *vs* 2.5 ± 1.7, respectively), suggesting a potential role of this ratio as an imaging biomarker for assessing the *in vivo* radiochemical stability of sEVs radiolabeled using this method.

### *Ex Vivo* Immunofluorescence Detection of PANC1 sEVs

**Figure 6:**
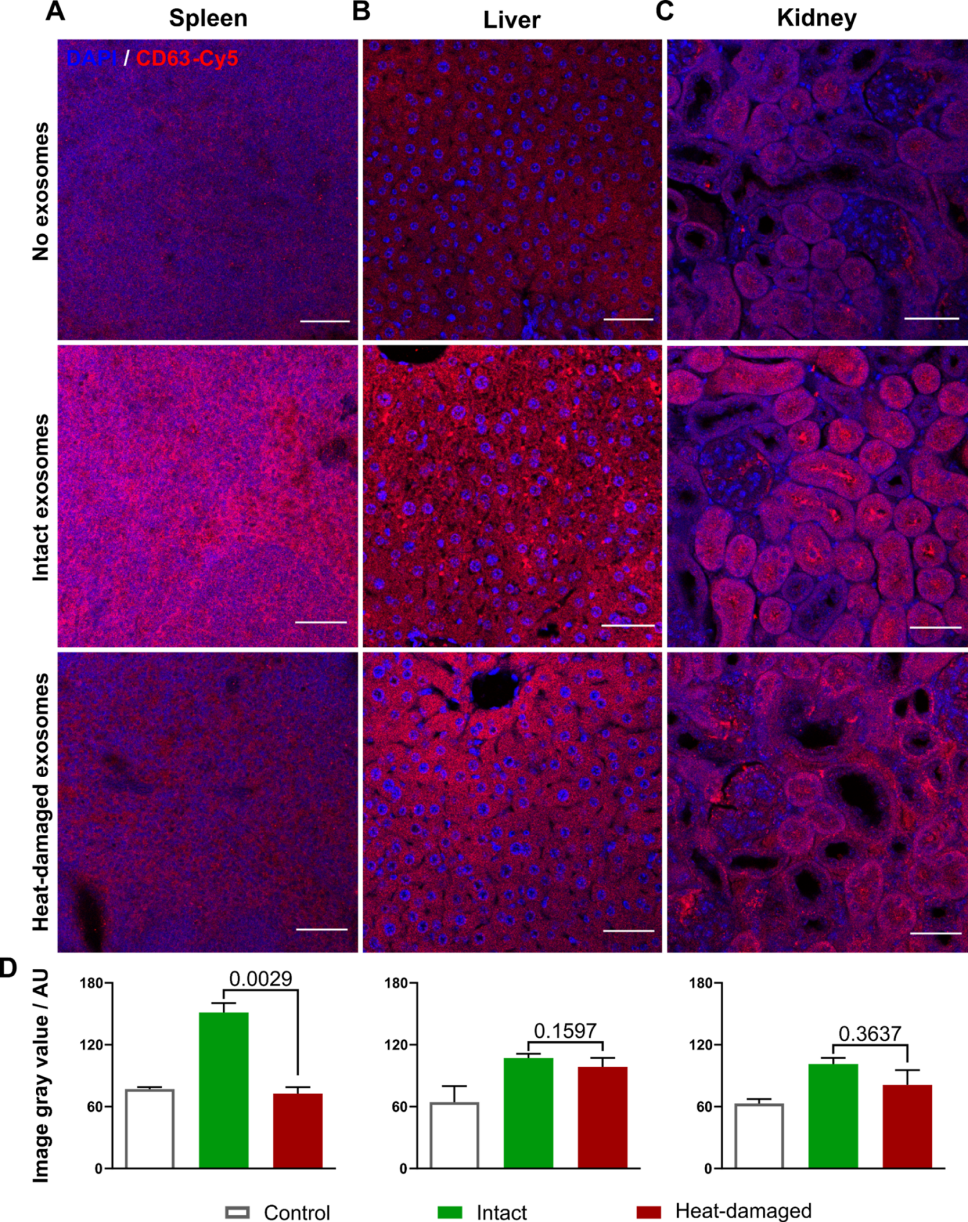
*Ex vivo* immunofluorescence detection of ^89^Zr-PANC1 sEVs. (A) Spleen, (B) liver, and (C) kidney sections from mice injected with no sEVs (control), intact ^89^Zr-PANC1 sEVs, and heat-damaged ^89^Zr-PANC1 sEVs were stained with anti-human CD63-Cy5 (red) and DAPI (blue) for cell nuclei. All samples were obtained, stained, and imaged using the same conditions/settings. Scale bar = 50 μm. (D) Random ROIs were drawn on the Cy5 images, and the signal intensity was calculated using ImageJ; data presented as mean ± SD of *n* = 3 and analyzed using one-way ANOVA.

To confirm that the ^89^Zr detected in the *in vivo* imaging and *ex vivo* biodistribution is from ^89^Zr-labeled PANC1 sEVs, immunofluorescence detection of some key organs was performed. Thus, the spleen, liver (highest sEV uptake), and kidney (very low sEV uptake) were probed for anti-human CD63-Cy5 to detect PANC1 sEVs (Figure 6). Tissues from C57BL/6j mice that had not been injected with sEVs served as the control for background fluorescence. Brighter fluorescence was observed in the spleen injected with intact PANC1 sEVs compared to heat-damaged sEVs, correlating with the PET imaging and *ex vivo* biodistribution data (Figure 6A). A similar finding was observed in the liver (Figure 6B), with increased presence of human CD63 in the intact sEV group, although the higher signal from the PET/*ex vivo* biodistribution experiments in this organ was not statistically significant. An interesting finding of this study, and our recent review on PET/SPECT imaging of EVs,^45^ is the presence of sEV renal excretion that we have previously suggested may be related to small EV fragments from fast EV metabolism/decomposition, as sEVs are much larger than the ∼55 kDa renal filtration threshold.^46^ Interestingly, the immunofluorescence microscopy data of the kidneys (Figure 6C) strongly suggest the presence of human CD63 proteins in PANC1 sEV-treated mice, as a strong fluorescence signal can be observed in the tubules of intact PANC1 sEV-treated mice. This finding could be due to either whole PANC1 sEVs present in kidney tubules, which would agree with the higher amount of the ^89^Zr signal from the biodistribution data, or CD63-containing fragments of sEVs that were able to pass through renal filtration.

For signal quantification, ROIs were drawn randomly to include areas of bright and weak fluorescence (Figure 6D). Spleen fluorescence was significantly higher for intact sEVs compared to heat-damaged sEVs, corresponding to both PET imaging and *ex vivo* biodistribution. Moreover, both the heat-damaged sEV fluorescence and control group fluorescence show a similar low signal. This further reinforces the previous proposal (Figure 5D) that the spleen uptake for ^89^Zr-labeled PANC1 sEVs can be used as an imaging biomarker to determine the sEV’s stability and quality. Correlating to *in/ex vivo* findings, there was no statistically significant difference between the intact and heat-damaged group for the liver and kidney. Although, according to the PET imaging and the biodistribution data, radioactivity detected in the liver is considerably higher than that detected in the kidneys, the fluorescence intensity level is very similar. As such, it can be proposed that once ^89^Zr-labeled exosomes are taken up by the liver, any ^89^Zr released from the vesicles is retained within this organ.

It is important to discuss the advantages and disadvantages of the radiolabeling method described in this report. Compared to other EV radiolabeling methods,^45^ [^89^Zr]Zr(oxinate)_4_ sEV radiolabeling benefits from radiochemical simplicity and low barriers for clinical translation, as this radiotracer is already being used in several preclinical and clinical trials for cell and liposomal nanomedicine tracking. The sEV RLY achieved is comparable to that reported for other sEV radiolabeling methods. Our data also strongly suggest that [^89^Zr]Zr(oxinate)_4_ sEV radiolabeling does not interfere with sEV membrane proteins, which is an advantage compared to methods that rely on covalent bond formation with membrane molecules (*e.g.,* bifunctional chelator-based) and hence are more likely to bind and affect their structure/function. We note, however, that further studies (*e.g.,* proteomics) would be required to fully validate this. We chose ^89^Zr (*t*_1/2_ = 3.3 d) due to its long half-life thus enabling PET tracking of sEVs for up to *ca*. >7 days. However, our *in vitro* stability studies showed *ca.* 25% release of ^89^Zr from radiolabeled sEVs, and thus *in vivo* PET-CT imaging was limited to 24 h to avoid analysis errors due to excessive levels of released free ^89^Zr. In terms of radiation dosimetry and potential clinical translation, indeed ^89^Zr may not be the radionuclide of choice if imaging is limited within this timeframe. It is worth noting, however, that compared to other radiometals such as ^64^Cu and ^52^Mn, ^89^Zr exhibits significantly better intravesicular/cellular retention.^31,47^

PET-CT imaging of ^89^Zr-PANC1 sEVs showed fast ^89^Zr uptake in the liver, spleen, and brain and suspected accumulation in LNs, which was supported by immunofluorescence imaging. The imaging data and high human-CD63 signal in the kidneys support the hypothesis that some populations of sEVs and/or sEV fragments can be cleared renally. We have also demonstrated that heat-damaged ^89^Zr-PANC1 sEVs show significant differences in spleen uptake, further supporting the key role this organ plays in the biodistribution of sEVs^48^ and leading us to propose the spleen/bone uptake ratio as an imaging biomarker for sEV stability when using [^89^Zr]Zr(oxinate)_4_ to radiolabel PANC1 sEVs.

## Conclusions

We have developed and optimized the synthesis of [^89^Zr]Zr(oxinate)_4_ and demonstrated that it allows simple, efficient, and direct labeling of sEVs. Using PANC1 sEVs as a model, our results demonstrated that sEVs retain their morphological characteristics following radiolabeling with [^89^Zr]Zr(oxinate)_4_ and also strongly suggest that surface biomolecules are not affected. *In vivo* PET-CT imaging in healthy mice showed that ^89^Zr-labeled sEVs are stable for 24 h and thus can reliably be tracked within this timeframe. The differential spleen:bone uptake ratio for intact *versus* heat-damaged ^89^Zr-PANC1 sEVs led to the proposition of using this parameter as an imaging biomarker for sEV stability when using this radiolabeling method. Further work will aim at understanding the nature of the extensive lymph node and brain ^89^Zr uptake and using PET imaging to support the development of sEVs as nanotherapeutics. We believe that this radiochemical tool will help the field to further investigate the *in vivo* behavior of sEVs and answer questions on their basic biology, supporting their applications as delivery vehicles, disease biomarkers (*e.g.,* identify metastatic niches), or as therapeutics.

## Experimental Procedures

### Synthesis of [^89^Zr]Zr(oxinate)_4_ (Method 1)

^89^Zr (10–100 MBq) in 1 M oxalic acid (PerkinElmer), diluted to 300 μL with deionized water (pre-treated with Chelex resin, 50–100 mesh size), was loaded onto a pre-conditioned QMA light cartridge (Sep-Pak, Waters) (conditioned with 5 mL of ethanol, 10 mL of saline, and 10 mL of deionized water). Trapped ^89^Zr^4+^ was eluted with 500 μL of 1 M HCl, and [^89^Zr]ZrCl_4_ was collected between 150 and 500 μL. [^89^Zr]ZrCl_4_ was dried at 60 °C under N_2_ in a Wheaton (V-bottom) glass vial, followed by addition of 80 μL of aqueous buffered oxine (8-hydroxyquinoline, 8HQ) solution containing 0.5 mg/mL 8HQ and 1 mg/mL Tween-80, and 1 M HEPES at pH 7.8 was added. [^89^Zr]Zr(oxinate)_4_ was then incubated at 4 °C or at RT for 10 min.

For ^89^Zr-control, a separate control kit was prepared with 1 M HEPES and 1 mg/mL Tween-80, neutralized to pH ∼ 7.8 with 10 M NaOH. The control kit was added to dry [^89^Zr]ZrCl_4_ and incubated at 4 °C for 10 min.

### Alternative Method for the Synthesis of [^89^Zr]Zr(Oxinate) (Method 2)

To aqueous [^89^Zr]ZrCl_4_, 40 μg of 8HQ in ethanol (3 M) was added and neutralized to pH ∼ 7.2 with 1 M NaHCO_3_. The ^89^Zr control was prepared by adding ethanol to [^89^Zr]ZrCl_4_ and neutralizing to pH ∼ 7.2 with 1 M NaHCO_3_.

### Radiochromatography

[^89^Zr]Zr(oxinate)_4_ complex formation was confirmed by iTLC; stationary phase = Whatman No 1 paper (GE healthcare) and mobile phase = 100% ethyl acetate. The chromatograms were analyzed on LabLogic Mini-Scan MS-1000F (Eckert & Ziegler) using a β detector probe and processed using Pearl software or on a Cyclone Plus Storage Phosphor imager (PerkinElmer) equipped with Optiquant software.

### Partition Coefficient Measurements—logD_7.4_ (PBS)

Lipophilicity of [^89^Zr]Zr(oxinate)_4_ was assessed using a biphasic solvent system of PBS in octanol. The [^89^Zr]Zr(oxinate)_4_ and control ^89^Zr (10–20 μL, 1 MBq) obtained by both formation methods were added to separate tubes, containing 500 μL of both PBS and octanol. Triplicate samples were prepared. The mixtures were vortexed at maximum speed for 3 min, followed by centrifugation at 16,000*g* for 3 min. Aliquots from each phase were transferred to separate Eppendorf tubes, and activities were measured using a gamma counter (Wallac Wizard 1282 CompuGamma, PerkinElmer).

### Cell Culture

For sEV isolation, all cells were cultured in cell media supplemented by 10% exo-depleted foetal bovine serum (FBS). FBS was depleted of exosomes or sEVs by ultracentrifugation at 100,000*g* for 18 h at 4 °C in a Beckman L60 ultracentrifuge with a SW41 Ti rotor (Beckman Coulter), followed by sterile filtration of the top two layers through a 0.22 μm PES membrane filter (Merck). MDA-MB-231.CD63-GFP, human metastatic breast cancer and PANC1, human metastatic pancreatic cancer cells were cultured in CELLine AD1000 bioreactor flasks (Wheaton) at 37 °C and in 5% CO_2_, as described by Mitchell *et al.*(49) Cells were cultured in 15 mL of low glucose DMEM and RPMI 1640, respectively, supplemented with 10% exo-depleted FBS, 1% penicillin–streptomycin, and 1% l-glutamine (all supplied by Sigma-Aldrich) in the bottom cell chamber, with 500 mL of the same medium as before, except that exo-depleted FBS was replaced with standard FBS, in the top reservoir chamber of the bioreactor flask. The cell supernatant was collected weekly and replaced with fresh exo-depleted cell media. Medium in the reservoir chamber was also replaced weekly. Immediately after collection, the supernatant was subjected to centrifugation at 500*g* for 5 min twice followed by at 2000*g* for 15 min, then filtration through a 0.22 μm PES filter. This filtered conditioned medium (CM) was stored at 4 °C for up to 6 weeks until used for sEV isolation.

### sEV Isolation

MDA-MB-231.CD63-GFP and PANC1 sEVs were isolated by following a protocol described previously.^16^ Briefly, 22.5 mL of CM was layered on 3 mL of 25% (w/w) sucrose cushion in D_2_O (Sigma-Aldrich) in a thick-walled polycarbonate centrifuge tube (Beckman Coulter) and ultracentrifuged (SW48 Ti rotor) at 100,000*g* for 1.5 h at 4 °C. The sucrose layer was transferred to another thick-walled centrifuge tube containing PBS, followed by another ultracentrifugation step (70.1 Ti rotor) at 100,000*g* for 1.5 h at 4 °C. Finally, the supernatant was discarded, and the sEV pellet was suspended in 200 μL of PBS and stored at 4 °C.

### Nanoparticle Tracking Analysis

The hydrodynamic diameter and concentration of sEVs were measured by NTA using NanoSight LM10, equipped with a 488 nm blue laser and NTA software v3.2 (Malvern Panalytical). The stock sample was diluted to achieve about 20–80 particles/viewing frame. Measurements were made in triplicates for 60 s, for up to three serial dilutions of the sample. Parameters used to capture and analyze data are as follows: screen gain = 2, camera level = 13, FPS = 25, viscosity = water, and detection threshold = 5.

### Cryo-Electron Microscopy

QUANTIFOIL R 2/2 carbon grids (mesh: Cu 300, #234901; Agar Scientific) were plasma discharged for 50 s at 30 SCCM gas flow in Nanoclean 1070 (Fischione instruments). Aliquots (3 μL) of non-radiolabeled or ^89^Zr-labeled PANC1 sEVs in PBS were deposited on the carbon grids in Vitrobot Mark IV (FEI). This was followed by blotting with standard Vitrobot filter paper (Agar Scientific) to remove excess liquid; blotting time = 2 s, wait time = 30 s, and blotting force = −2. The grids were then plunge frozen in liquid ethane (−188 °C) and maintained in liquid N_2_ (−196 °C) in a grid box and transferred into a cryo-transfer holder. CryoEM was performed on TECNAI 12 G^2^ (FEI) connected to a TemCam-F216 camera and Temmenu v4 software (Tietz Video & Image Processing Systems GmbH, Germany). Parameters used to capture images are as follows: electron acceleration = 120 kV, magnification = 42,000×, acquisition time = 1 s, defocus = −2.5 to −3 μm, and spot size = 5. To minimize radiation damage during localization of sEVs, grids were visualized using the low-dose mode.

### BCA Protein Assay

The protein content of the sEVs was analyzed in duplicates of up to three serial dilutions using Pierce Rapid Gold BCA protein assay (Thermo Fisher), according to the manufacturer’s microplate protocol. Absorbance was measured at 480 nm on SPECTROstar Nano (BMG Labtech).

### Dot Blot

For membrane markers, 40 μL of sEVs (1 × 10^10^ particles/mL) and for intraluminal and negative markers, 1 × 10^10^ particles in 40 μL were spotted on nitrocellulose membranes (0.45 μm; Bio-Rad) and incubated at RT for 1 h in blocking buffer (3% milk in TBS-T). Mouse anti-human CD63 (BioLegend #353013), CD81 (BioLegend #349520), CD9 (BioLegend #312102), and Calnexin (GeneTex #GTX629976-S) antibodies at 0.5 μg/mL and Alix (Cell Signalling Technology #2171S) at 0.2 μg/mL in blocking buffer were added to separate membranes and incubated overnight at 4 °C. Staining was performed with an HRP-conjugated goat anti-mouse IgG antibody (1:10,000 dilution in blocking buffer; BioLegend #405306) for 1 h at RT. A chemiluminescence signal was detected using a SuperSignal West Atto Ultimate Sensitivity substrate (Thermo Fisher), imaged on iBright FL1000 (Invitrogen) or developed on a CL-Exposure film (Thermo Fisher).

### Bead-Assisted Flow Cytometry

The protocol for bead-assisted flow cytometry for sEVs was adapted from Thery *et al.*(50) Unlabeled or ^89^Zr-labeled PANC1 sEVs (intact or heat-damaged) at a concentration of 1 × 10^10^ sEVs in 40 μL of PBS were incubated with 10 μL of aldehyde/sulfate latex beads (3.9 μm, 4% w/v; Molecular Probes) for 15 min at RT. 10 μM BSA was added to the sEV-bead mixture and incubated for 15 min at RT. 1 mL of PBS was added and incubated for further 75 min at RT on an orbital rotator. The beads were pelleted by centrifugation for 5 min at 600*g*, re-suspended with 1 mL of 100 mM glycine in PBS, and incubated for 30 min at RT. The beads were washed twice with 2% FBS in PBS (FBS/PBS). Aliquots of the sEV-bead suspension were incubated with 1 μg of mouse anti-human CD63 (BioLegend #353013), CD81 (BioLegend #349520), and CD9 (BioLegend #312102) antibodies in separate tubes or with no primary antibody (2° only control) for 40 min at 4 °C. The beads were washed once, re-suspended in FBS/PBS, and incubated with goat anti-mouse AlexaFluor 647 (0.5 μg/mL; BioLegend #405322) for 40 min at 4 °C, covered in foil. Finally, the beads were washed and suspended in 200 μL of FBS/PBS for flow cytometry analysis on FACS Melody (BD Biosciences), and the data were analyzed on FlowJo v10. The 2° only population was used for gating control.

### Radiolabeling of sEVs

MDA-MB-231.CD63-GFP sEVs, *ca.* 1 × 10^10^ vesicles, and *ca.* 1 × 10^11^ PANC1 sEVs in 160 μL of PBS were incubated with 20 μL of [^89^Zr]Zr(oxinate)_4_ or ^89^Zr control for 20 min at 37 °C with frequent shaking, followed by addition of 100 μL of 1% DFO (deferoxamine mesylate salt, ≥92.5%; Sigma) in PBS to trap any unbound ^89^Zr. Radiolabeled sEVs were purified from an unchelated radiotracer by SEC using Exo-spin mini-HD columns (Cell Guidance Systems) or self-prepared Sepharose CL-2B resin (GE Healthcare). The resin was self-packed under gravity into empty G-25 MiniTrap columns (GE Healthcare). The reaction mixture was loaded onto the column, and the purified sample was eluted using the manufacturer’s protocol for either mini-HD or minitrap columns. Radioactivity of the eluate and the column was measured using a gamma counter to calculate RLY.

### Heat Damaging of ^89^Zr-PANC1 sEVs

After radiolabeling, sEVs were damaged by a ×2 heat/cool cycle—heating to 90 °C for 20 min followed by incubation in ice for 10 min, repeated once more. Expression of sEV marker proteins after heat damage was analyzed by bead-assisted flow cytometry. To evaluate damage, sEVs were passed through an Exo-spin mini-HD column for characterization by NTA, BCA protein assay, and RLY.

### *In Vitro* Stability of ^89^Zr-PANC1 sEVs in PBS

^89^Zr-PANC1 sEVs (intact or heat-damaged) were incubated in PBS at 37 °C for up to 72 h (*n* = 2 in duplicate for up to 24 h, *n* = 1 in duplicate thereafter). Stability was assessed by iTLC; stationary phase = Whatman No 1 paper (GE healthcare) and mobile phase = 10 mM EDTA at pH 6.^51^ The chromatograms were analyzed on LabLogic Mini-Scan MS-1000F (Eckert & Ziegler) using a β detector probe and processed using Pearl software. *In vitro* stability was calculated by comparing the radioactivity associated at *R*_f_ = 0 compared to the rest of the chromatogram.

### *In Vitro* Cell Uptake of ^89^Zr-PANC1 sEVs

Uptake of ^89^Zr-PANC1 sEVs was assessed using four different cell types: (1) PANC1, (2) HEK293T, (3) MDA-MB-231, and (4) DU145. In a 24-W plate, 50,000 cells/well were seeded and maintained in serum-supplemented growth media at 37 °C and in 5% CO_2_. After 24 h, the ^89^Zr-PANC1 sEVs, [^89^Zr]Zr(oxinate)_4_, or ^89^Zr control were added to each cell type in triplicate. Cell uptake was assessed at 4 h. Radioactivity of the supernatant and the cells was measured separately, and the uptake of the radiotracer was calculated.

### PET-CT Imaging

Animal studies were carried out in accordance with the UK Home Office regulations under The Animals (Scientific Procedures) Act 1986. Immunocompetent C57BL/6j male mice (8–10 weeks) were anaesthetized with 2–2.5% isoflurane in 100% oxygen. ^89^Zr-PANC1 sEVs (0.2–1 MBq, ∼1 × 10^10^ sEVs in 104–140 μL of PBS/mouse), either intact (*n* = 4) or heat-damaged (*n* = 3), were injected intravenously *via* the tail vein at *t* = 0. For free ^89^Zr^4+^ biodistribution, [^89^Zr]ZrCl_4_ neutralized with 1 M NaHCO_3_ (0.8–1.2 MBq in 68–130 μL) was injected intravenously.

PET-CT imaging was performed on a nanoScan PET-CT preclinical imaging system (Mediso Medical Imaging System) using an air-heated standard single bed or a four-bed mouse hotel;^52^ anesthesia was maintained throughout the scans. PET imaging was started at *t* = 0.5 h for 2 h and at *t* = 24 h for 1 h followed by a CT scan. All PET/CT data were reconstructed in Nucline v.0.21 (Mediso Medical Imaging System) using Monte Carlo-based Tera-Tomo 3D PET reconstruction (400–600 keV energy window, 1–3 coincidence mode, and 4 iterations and 6 subsets) at an isotropic voxel size of 0.4 mm; images were corrected for scatter attenuation and were decay corrected to the time of injection. Reconstructed images were analyzed using VivoQuant (inviCRO Inc).

At the end of the imaging session at *t* = 24 h, mice were culled by cervical dislocation while under anesthesia. Blood, urine, and organs of interest were collected and weighed for the *ex vivo* biodistribution study. Standards of the injected radiotracer were prepared by serial dilutions. These standards along with the collected tissues were gamma counted to calculate the percentage injected dose (%ID/g).

### Immunofluorescence Detection

Following *in vivo* imaging, the spleen, liver, and kidneys were fixed in 10% neutral buffered formalin at 4 °C for up to 48 h, maintained in 70% ethanol until radioactivity decayed, and embedded in paraffin. Organ sections (5 μm) were de-paraffinized, and antigen retrieval was performed in 10 mM citrate buffer (pH 6) with 0.1% Tween-20 at 100 °C for 20 min. Sections were blocked with 5% goat serum and 1% BSA for 1 h at RT and incubated in a rabbit anti-human CD63 (EPR5702, 1.9 μg/mL; Abcam, # ab134045) antibody overnight at 4 °C. Tissues were then stained with Cy5 (3 μg/mL; Jackson ImmunoResearch, #111-175-144) for 1 h at RT and mounted using Fluoroshield DAPI (Sigma). Confocal microscopy was performed on an Eclipse Ti-E A1 inverted confocal microscope with a Plan Apo λ 20× objective (Nikon), and images were analyzed on ImageJ. For signal quantification, images were split into separate channels—red and blue, and random ROIs were on the red channel grayscale image for Cy5 and quantified using the “analyze” and “measure” tool on ImageJ.

### Statistical Analysis

All numerical data were analyzed on GraphPad Prism 8 or Microsoft Excel 2016. All values are given in one decimal place. Data are presented as mean ± standard deviation (SD), unless stated otherwise. Unless specified, Student’s unpaired *t*-test was used to calculate statistical differences between groups with the *P* value < 0.05 considered significant. Exact significance values are reported in each figure.

## Supporting Information Available

Characterization of the ^89^Zr control and comparison of [^89^ZR]Zr(oxinate)_4_ radiochemical properties, evaluation of Sepharose-based SEC systems, effect of DFO and Tween-80 on the radiolabeling of sEVs, radiochemical stability of ^89^Zr-PANC1 sEVs, validation of ^89^Zr-PANC1 damage, ^89^Zr-PANC1 sEV signal, heat-damaged ^89^Zr-PANC1 sEVs’ signal, ex vivo distribution, and biodistribution data (PDF)
