# Supplementary material for: PET Imaging of Small Extracellular Vesicles via [89Zr]Zr(oxinate)4 Direct Radiolabeling
Source: Bioconjug Chem. 2022 Feb 28;33(3):473–85. doi: 10.1021/acs.bioconjchem.1c00597 (PMC8931726; doi:10.1021/acs.bioconjchem.1c00597)
Supplement: Supplementary file 1 — bc1c00597_si_001.pdf [file bc1c00597_si_001.pdf]

Supporting Information

PET imaging of small extracellular vesicles *via* [^{89}Zr]Zr(oxinate)$_4$ direct radiolabelling

*Azalea A. Khan [a], Francis Man [a,b], Farid N. Faruqu [b], Jana Kim [a], Fahad Al-Salemee [a], Amaia Carrascal-Miniño [a], Alessia Volpe [a], Revadee Liam-Or [b], Paul Simpson [c], Gilbert O. Fruhwirth [a], Khuloud T. Al-Jamal [b], and Rafael T. M. de Rosales [a]**

*E-Mail: rafael.torres@kcl.ac.uk

[a] Department of Imaging Chemistry and Biology, School of Biomedical Engineering and Imaging Sciences, King's College London, St. Thomas' Hospital, London, SE1 7EH.

[b] Institute of Pharmaceutical Sciences, School of Cancer & Pharmaceutical Sciences, King's College London, Franklin Wilkins Building, London, SE1 9NH.

[c] Electron Microscopy Centre, Department of Life Sciences, Faculty of Natural Sciences, Imperial College London, Flowers Building, London, SW7 2AZ.

Supplementary figures

Figure S1

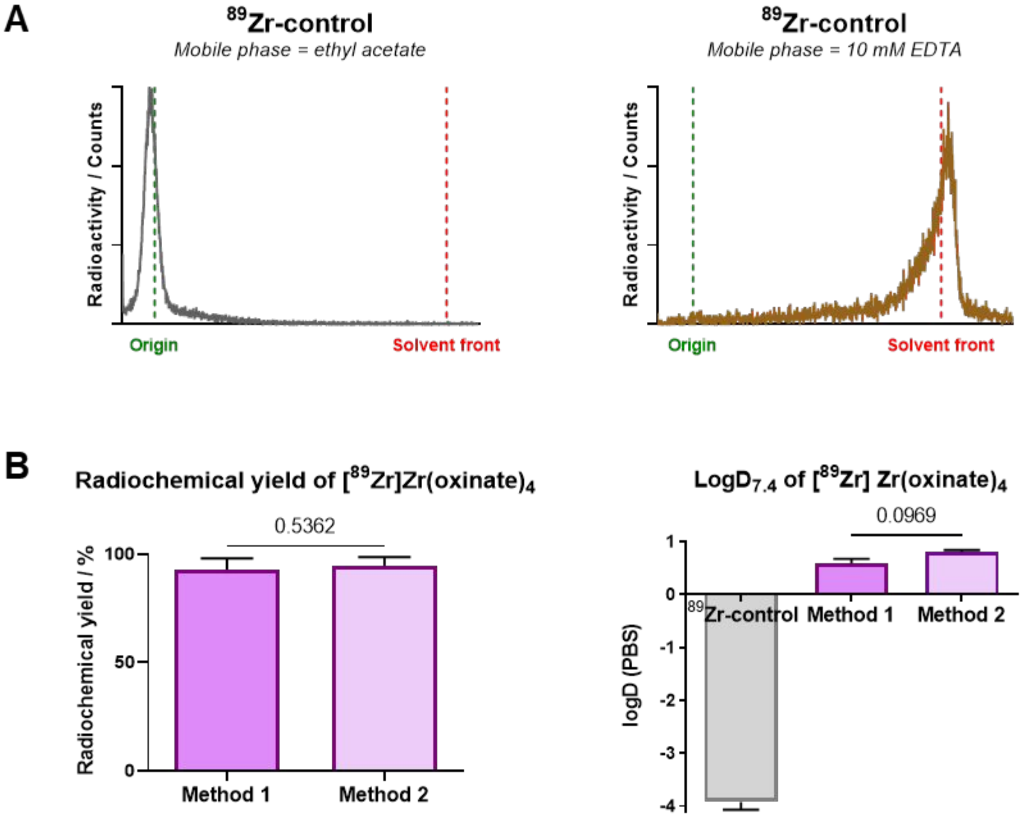

Figure S1: Characterisation of ^{89}Zr-control and comparison of [^{89}Zr]Zr(oxinate)$_4$ radiochemical properties synthesised by method 1 and method 2.

A) Radiochromatogram of ^{89}Zr-control showing peak *(left)* at R_f = 0 (origin) when using ethyl acetate, and *(right)* at R_f = 1 (solvent front) when using 10 mM EDTA as the mobile phase. **B)** *(left)* Radiochemical yield of [^{89}Zr]Zr(oxinate)$_4$ formulations (n = 5), and *(right)* LogD$_{7.4}$ (PBS) of control ^{89}Zr and [^{89}Zr]Zr(oxinate)$_4$ (n = 3), analysed by one-way ANOVA with Tukey's correction for multiple comparisons. Data presented as mean ± SD.

Figure S2

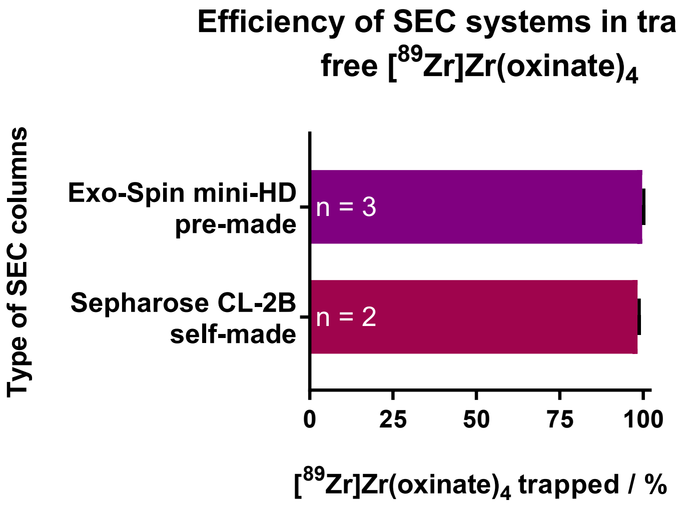

Figure S2: Evaluation of Sepharose-based SEC systems in trapping of $[^{89}Zr]Zr(oxinate)_4$.

$[^{89}Zr]Zr(oxinate)_4$ was passed through the two Sepharose-based SEC columns in the absence of any sEVs, the n numbers are specified in the figure for each column. The efficiency was determined by loading only $[^{89}Zr]Zr(oxinate)_4$, after 5 min incubation with DFO, and the radioactivity retained in the column was measured as a fraction of the total radioactivity loaded.

Figure S3

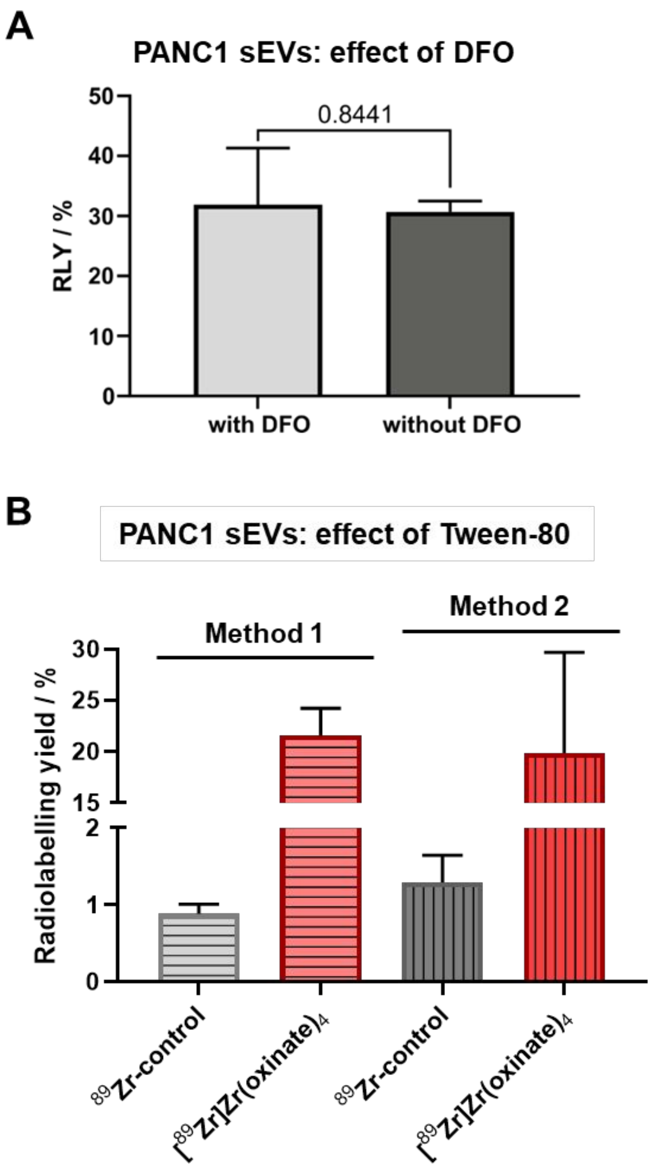

Figure S3: Effect of DFO and Tween-80 on the radiolabelling of sEVs.

(A) The addition of DFO during the purification process does not affect the RLYs obtained. After $1x10^{12}$ PANC1 sEVs were incubated with $[^{89}Zr]Zr(oxinate)_4$ for 20 min at 37 °C, the reaction mixture was incubated for another 5 min with (n = 3) or without (n= 2) DFO, and purified by SEC. Data given as mean ± SD of the specified n number and analysed by Welch's t-test. **(B)** PANC1 sEVs radiolabelling yields after reacting an equal number of PANC1 sEVs ($1x10^{11}$) with ^{89}Zr-control and $[^{89}Zr]Zr(oxinate)_4$, synthesised using both Method 1 and Method 2 (n = 2–3). See methods section for details about the two $[^{89}Zr]Zr(oxinate)_4$ synthesis methods.

Figure S4

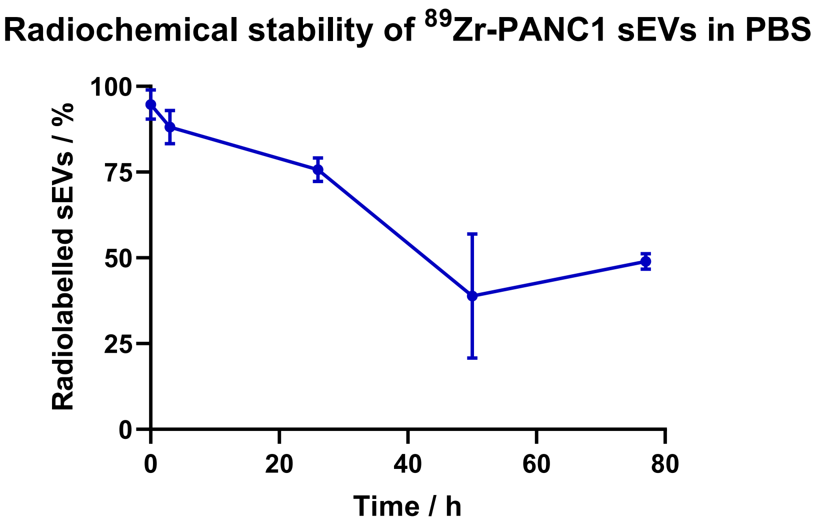

Figure S4: Radiochemical stability of ^{89}Zr-PANC1 sEVs.

^{89}Zr-PANC1 sEVs were incubated in PBS at 37°C and stability was analysed by iTLC (n = 2–3 in duplicate). iTLC: stationary phase = Whatman No1 paper, mobile phase = 10 mM EDTA at pH 6.

Figure S5

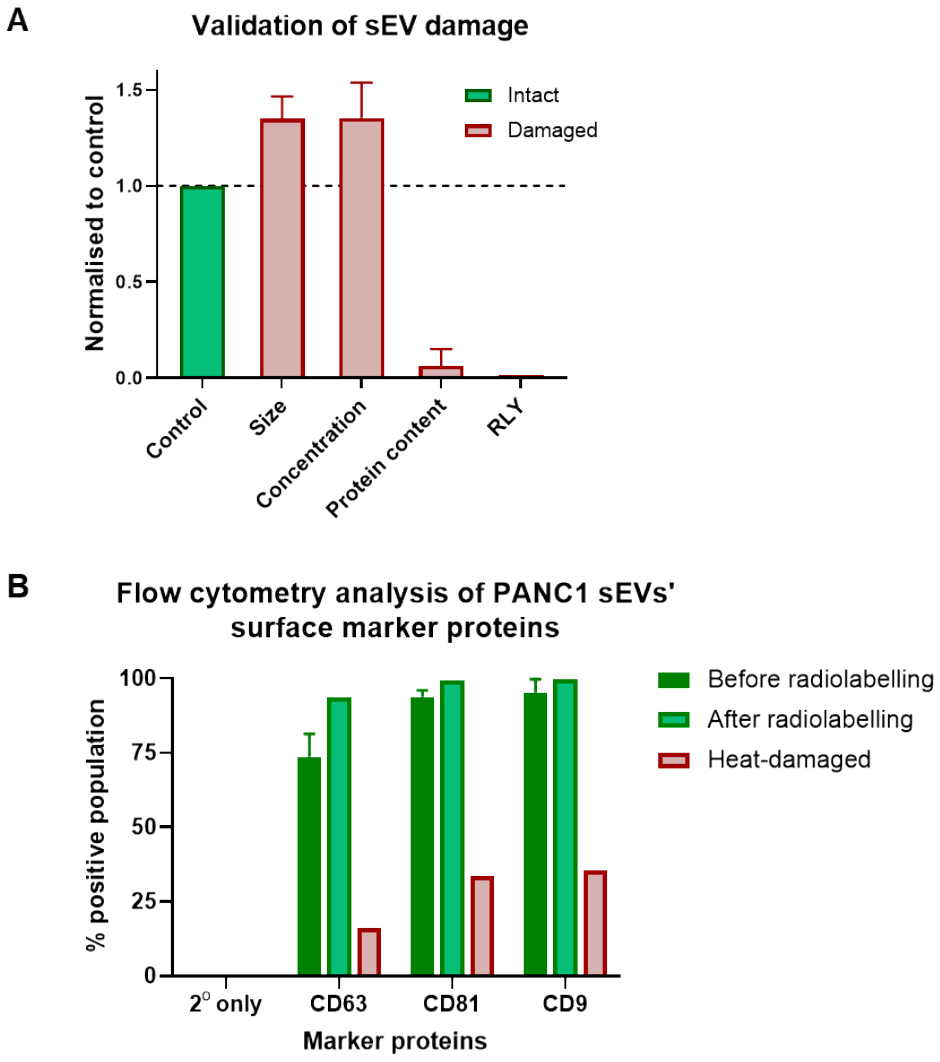

Figure S5: Validation of ^{89}Zr-PANC1 sEV damage.

After radiolabelling, sEVs were subjected to heat (90 °C for 20 min) and cold (0 °C for 10 min) twice, and purified by SEC. **(A)** Diameter, concentration, protein content and radiolabelling yield of the sEVs were compared to control (intact sEVs). Data presented here were normalised to the control group and given as mean ± SD of n = 2. **(B)** For protein marker expression, after heat-damage, sEVs were analysed by flow cytometry. For the three marker proteins, CD63, CD81 and CD9, % of positive population data was analysed. Data presented for PANC1 sEVs before radiolabelling (n = 2), after radiolabelling (n = 1) and heat-damaged (n = 1).

Figure S6

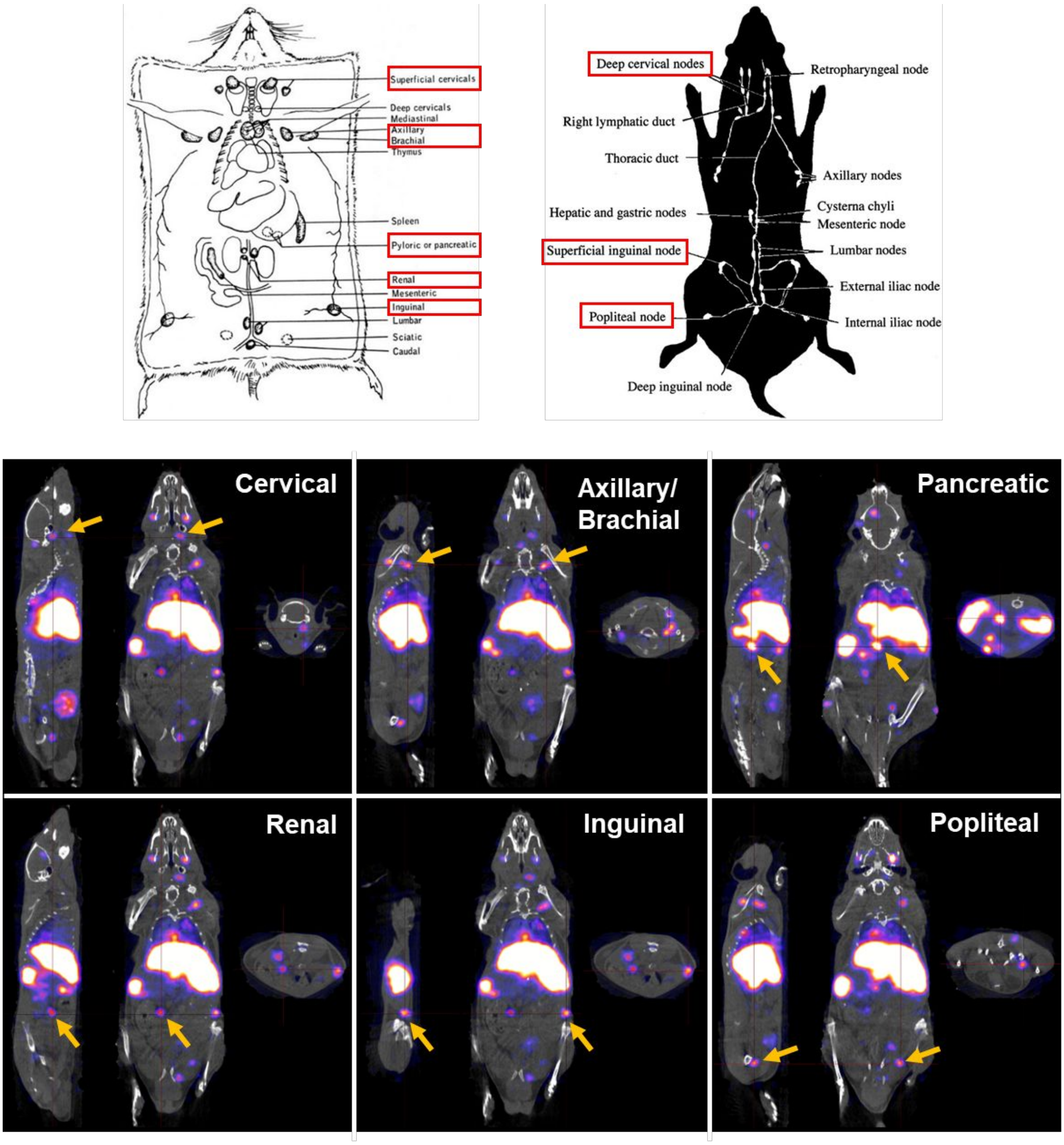

Figure S6: ^{89}Zr-PANC1 sEVs signal in several lymph nodes.

PET-CT images of a mouse injected with intact ^{89}Zr-PANC1 sEVs and showing apparent uptake in lymph nodes, based on well-documented anatomical location of mouse lymph nodes.[1-2]

Figure S7

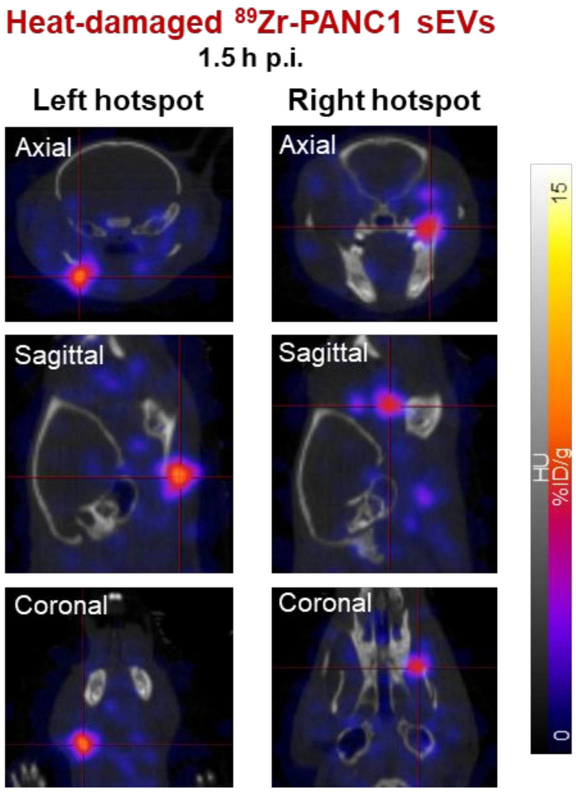

Figure S7: Heat-damaged ^{89}Zr-PANC1 sEVs signal in the head.

PET-CT images (axial, sagittal and coronal slices) of a mouse injected with heat-damaged ^{89}Zr-PANC1 sEVs (MIP of the same mouse: **Fig. 5Aii)**; confirming that the two hot spots observed in the MIP PET image are not in the brain and are likely head and neck lymph nodes.[3]

Figure S8

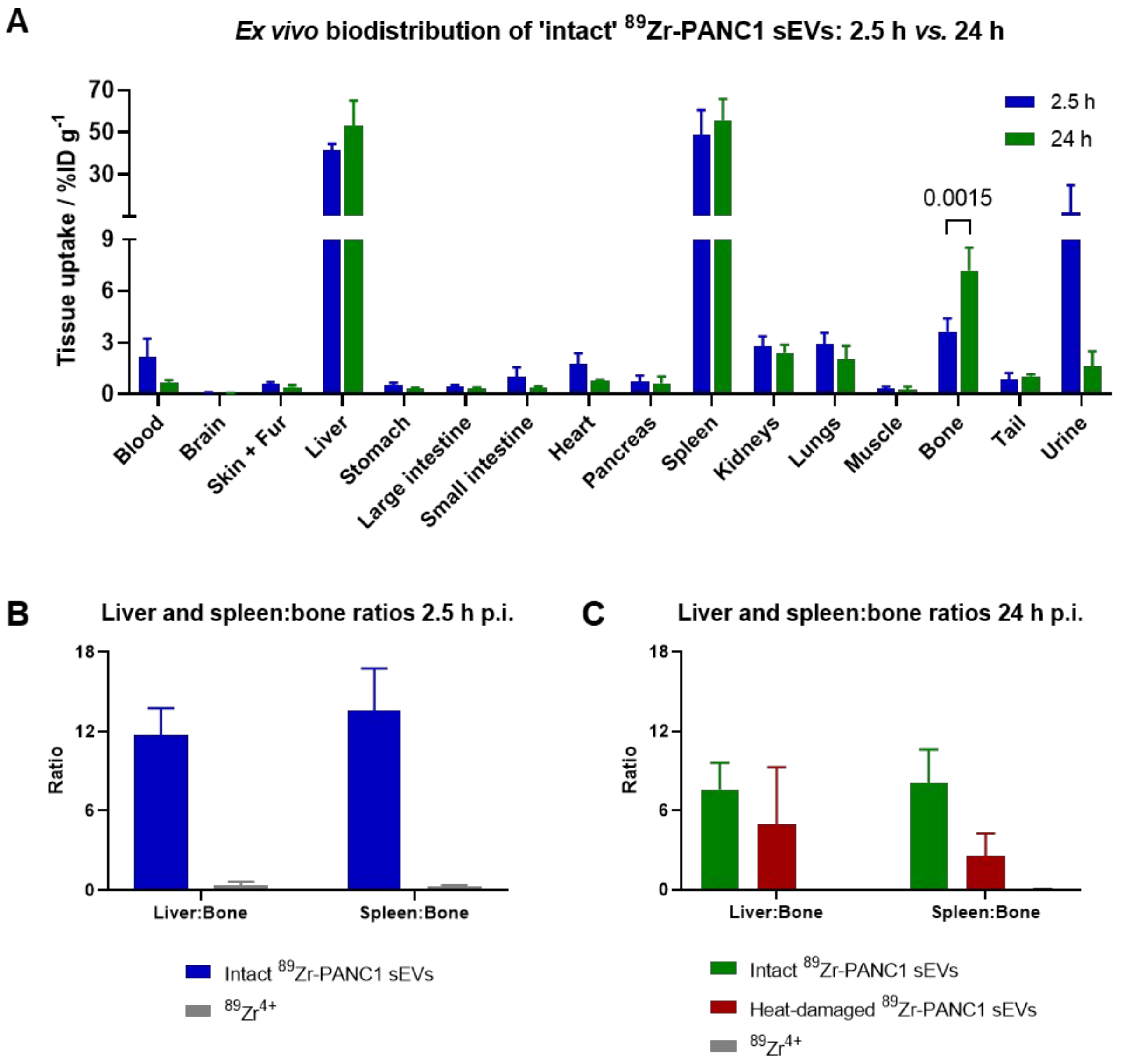

Figure S8: *Ex vivo* biodistribution of intact ^{89}Zr-PANC1 sEVs.

(A) *Ex vivo* biodistribution showing uptake of "intact" ^{89}Zr-PANC1 sEVs at 2.5 h (n = 5) and 24 h (n = 4) post iv. injection; data given as mean ± SD; statistical significance was calculated using Student's unpaired t-test. Ratio of liver:bone uptake and spleen:bone uptake **(B)** 2.5 h and **(C)** 24 h post iv. injection; data given as the geometrical mean ± SD.

Supplementary tables

Table S1

Table S1: Biodistribution data (%ID g^{-1}) for intravenously administered intact ^{89}Zr-PANC1 sEV and heat-damaged ^{89}Zr-PANC1 sEV 24 h post injection.

Organs	^{89}Zr Uptake (%ID g^{-1})		
	**Intact ^{89}Zr-PANC1 exosome** (n = 4)	**Heat-damaged ^{89}Zr-PANC1 exosome** (n = 3)	**$^{89}Zr^{4+}$** (n = 4)
Blood	0.68 ± 0.14	0.60 ± 0.49	2.34 ± 0.25
Brain	0.05 ± 0.02	0.06 ± 0.05	0.18 ± 0.04
Skin + Fur	0.41 ± 0.11	0.45 ± 0.40	1.99 ± 0.37
Liver	53.25 ± 11.75	36.27 ± 16.91	2.30 ± 0.30
Stomach	0.32 ± 0.07	0.27 ± 0.25	0.59 ± 0.13
Large intestine	0.35 ± 0.06	0.41 ± 0.36	0.66 ± 0.09
Small intestine	0.38 ± 0.07	0.43 ± 0.43	1.17 ± 0.13
Heart	0.79 ± 0.04	0.37 ± 0.28	1.37 ± 0.17
Pancreas	0.64 ± 0.39	0.35 ± 0.35	1.16 ± 0.25
Spleen	55.66 ± 10.24	20.09 ± 7.51	2.16 ± 0.42
Kidneys	2.39 ± 0.47	1.24 ± 0.56	2.26 ± 0.30
Lungs	2.04 ± 0.77	3.59 ± 4.68	2.34 ± 0.58
Muscle	0.27 ± 0.17	0.15 ± 0.15	0.98 ± 0.71
Bone	7.19 ± 1.34	10.47 ± 8.43	41.98 ± 3.04
Tail	1.04 ± 0.11	1.18 ± 0.91	8.26 ± 1.83
Urine	1.63 ± 0.85	0.37 ± 0.20	1.38 ± 0.57
Liver-to-Bone ratio	7.55 ± 2.07	4.92 ± 4.36	0.06 ± 0.01
Spleen-to-Bone ratio	8.06 ± 2.55	2.56 ± 1.70	0.05 ± 0.01

Table S2

Table S2: Biodistribution data (%ID) for intravenously administered intact ^{89}Zr-PANC1 sEV and heat-damaged ^{89}Zr-PANC1 sEV 24 h post injection for whole organs.

	**^{89}Zr Uptake (%ID) at 24 h**		
**Organs**	**Intact ^{89}Zr-PANC1 sEV** (n = 4)	**Heat-damaged ^{89}Zr-PANC1 sEV** (n = 3)	**Free $^{89}Zr^{4+}$** (n = 4)
Brain	0.02 ± 0.01	0.03 ± 0.03	0.08 ± 0.01
Skin + Fur *(both ears)*	0.08 ± 0.04	0.07 ± 0.06	0.20 ± 0.04
Liver	58.76 ± 9.63	40.14 ± 12.73	2.46 ± 0.55
Stomach	0.12 ± 0.02	0.08 ± 0.08	0.26 ± 0.02
Large intestine	0.15 ± 0.04	0.25 ± 0.17	0.61 ± 0.09
Small intestine	0.40 ± 0.17	0.39 ± 0.41	0.82 ± 0.20
Heart	0.14 ± 0.03	0.07 ± 0.05	0.17 ± 0.02
Pancreas	0.02 ± 0.01	0.02 ± 0.02	0.04 ± 0.01
Spleen	5.33 ± 1.64	1.54 ± 0.64	0.12 ± 0.03
Kidneys	0.86 ± 0.09	0.49 ± 0.19	0.64 ± 0.14
Lungs	0.32 ± 0.11	0.61 ± 0.79	0.37 ± 0.05
Muscle *(from one hind leg)*	0.06 ± 0.02	0.03 ± 0.02	0.25 ± 0.18
Bone *(tibia & fibula of one hind leg)*	0.39 ± 0.06	0.51 ± 0.42	2.22 ± 0.28
Tail	0.55 ± 0.06	0.64 ± 0.47	4.44 ± 1.20
Urine *(collected from full bladder)*	0.22 ± 0.12	0.10 ± 0.04	0.15 ± 0.06

References

1. Hummel, K.; Richardson, F.; Fekete, E., Anatomy. In *Biology of the Laboratory Mouse*, 2nd ed.; Green, E., Ed. Dover Publications: New York, 1968.
2. Kobayashi, H.; Kawamoto, S.; Star, R. A.; Waldmann, T. A.; Tagaya, Y.; Brechbiel, M. W., Micro-magnetic Resonance Lymphangiography in Mice Using a Novel Dendrimer-based Magnetic Resonance Imaging Contrast Agent. *Cancer Res.* **2003,** *63* (2), 271-276.
3. Lohrberg, M.; Wilting, J., The lymphatic vascular system of the mouse head. *Cell Tissue Res.* **2016,** *366* (3), 667-677.
